# Trends, Disparities, and Outcomes in Time to Treatment Initiation in Patients With Early‐Onset Colorectal Cancer

**DOI:** 10.1002/jgh3.70480

**Published:** 2026-10-08

**Authors:** Imran Qureshi, Charmi Patel, Muhammad Ali Ibrahim Kazi, Jackson Edwards, Hamza Khan, Kaveh Hajifathalian, Ahmed Al‐Khazraji

**Affiliations:** ^1^ Division of Gastroenterology, Hepatology and Nutrition University of Pittsburgh Medical Center Pittsburgh Pennsylvania USA; ^2^ Department of Internal Medicine Rutgers New Jersey Medical School Newark New Jersey USA; ^3^ Department of Internal Medicine Luminis Health Anne Arundel Medical Center Annapolis Maryland USA; ^4^ School of Medicine Rutgers New Jersey Medical School Newark New Jersey USA; ^5^ Division of Gastroenterology and Hepatology Rutgers New Jersey Medical School Newark New Jersey USA

**Keywords:** colorectal cancer, healthcare disparities, predictors of treatment delay, time to treatment initiation

## Abstract

**Aims:**

The incidence of early‐onset colorectal cancer (EOCRC) is on the rise globally. It is well documented in the literature that increased time to treatment initiation (TTI) results in higher mortality rates in malignancies. We conducted this study to explore trends, disparities, and outcomes in TTI in patients with EOCRC.

**Methods:**

We conducted a retrospective cohort study using the National Cancer Database. We identified 56 333 patients less than 45 years of age who were diagnosed with colorectal cancer between 2004 and 2021 who had data on TTI. We calculated the average annual percentage change (AAPC) in TTI and assessed predictors of treatment delay and the degree to which they are associated with delays using logistic and linear regression, respectively. A Cox proportional hazards model was fit for each stage to assess 5‐year, 10‐year, and overall mortality.

**Results:**

The overall AAPC was 1.50% (95% CI: 1.40–1.60, *p* < 0.001), with all variables showing positive AAPCs, but these varied significantly by sociodemographic factors. Sex, race, ethnicity, education, insurance, and comorbidity burden were predictors of treatment delay. There was increased overall mortality in Stage 2 (aHR = 1.48, 95% CI: 1.30–1.69, *p* < 0.001) and Stage 3 disease (aHR = 1.12, 95% CI: 1.03–1.21, *p* = 0.007) and decreased overall mortality in Stage 4 disease (aHR = 0.84, 95% CI: 0.79–0.88, *p* < 0.001) in patients who experienced treatment delays.

**Conclusions:**

Treatment delays are increasingly common and strongly associated with insurance status, socioeconomic disadvantage, and race, with clinically meaningful delays of several days, while patients with advanced disease are prioritized for faster treatment. These provide public health stakeholders with important insights into inequities in the treatment of EOCRC.

## 1 Introduction

Colorectal cancer (CRC) is the third most common and second most lethal cause of cancer worldwide [1]. While the overall incidence has declined, early‐onset colorectal cancer (EOCRC) has experienced a striking rise in recent decades [2]. CRC is the most common cause of cancer‐related death in men and the second most common cause of cancer‐related death in women under 50 [3]. Compared to individuals born in 1950, the age‐specific risk for colon cancer is double and for rectal cancer is quadruple in individuals born in 1990 [4]. By 2030, early‐onset cases are expected to account for one in 10 cases of colon cancer and one in four cases of rectal cancer [5]. This alarming trend is likely driven by a combination of environmental, genetic, and lifestyle factors [6, 7, 8]. Younger patients also tend to receive more aggressive treatment than their older counterparts [9]. Furthermore, time to treatment initiation (TTI) remains a critical determinant of outcomes, and delays can be particularly detrimental in younger patients with potentially curable disease [10, 11].

Generally, there has been an increase in TTI for most cancers [12]. The COVID‐19 pandemic further amplified this, as limited resources have lengthened the TTI among many cancer populations, more notably among the underserved. This prolonged TTI has resulted in higher all‐cause mortality rates [13]. It is important to note that those among lower socioeconomic status, marginalized communities, and specific geographic locations lacking high‐quality cancer care are more likely to present with metastatic disease and have worse outcomes [10, 14, 15]. These advanced disease presentations at the time of diagnosis are strongly associated with higher morbidity and mortality rates [16]. For this reason, early diagnosis and prompt initiation of treatment for EOCRC should, in theory, be critical to improving outcomes among all individuals with cancer. That being said, according to the American Cancer Society, the median time to initial clinical presentation after symptom onset was twice as long in patients with EOCRC compared with older patients [17]. Given the concerning rise in EOCRC rates, it is crucial to explore the accessibility, barriers, and advantages of early diagnosis and treatment for patients with EOCRC. However, data in this field remains limited. Therefore, this study aimed to assess trends, identify disparities, and gauge TTI outcomes among EOCRC patients, reflecting the growing burden of this disease.

## 2 Materials and Methods

### 2.1 Data Source

We conducted a retrospective cohort study using the National Cancer Database's (NCDB) Participant User File (PUF). The NCDB is an oncology‐specific database managed by the American College of Surgeons Commission on Cancer and the American Cancer Society. The NCDB annually captures over 70% of new cancer diagnoses in the US from over 1500 accredited facilities, which are abstracted by trained reviewers [18]. The NCDB PUFs are Health Insurance Portability and Accountability Act (HIPAA) compliant data files containing de‐identified hospital‐level patient data from Commission on Cancer (CoC) accredited facilities. NCDB PUFs are available to investigators associated with CoC‐accredited cancer programs [19] through an application process. This study was exempted from Institutional Review Board review because it contains de‐identified data. This manuscript is written in adherence with the Strengthening the Reporting of Observational Studies in Epidemiology (STROBE) reporting guideline.

### 2.2 Cohort Selection

We queried the NCDB 2022 PUF for patients diagnosed with CRC between 2004 and 2021 using International Classification of Diseases for Oncology, Third Edition (ICD‐O‐3) topography codes C180‐C189, C199, and C209 (*n* = 1 905 918). This PUF did not report mortality among patients diagnosed with CRC in 2022, so we excluded those patients (*n* = 104 174). We defined EOCRC as patients with CRC who were younger than 45 years and excluded those 45 years of age or older (*n* = 1 686 466). TTI was defined as the number of days from histologic diagnosis of CRC to receipt of any therapy. We excluded patients with a TTI of less than a week, as these were likely emergent cases. We also excluded patients who had treatment initiation more than 365 days after diagnosis, given the small sample size and validity concerns, as this was likely a coding error, such as an incorrect time of diagnosis or treatment initiation [20, 21]. Patients who did not receive any treatment or had missing data on treatment initiation were excluded from the primary analysis. Because treatment delay was defined as the time from diagnosis to treatment initiation, patients who died before treatment initiation could not be classified into delay categories and were therefore excluded from the treatment delay analyses. After applying our inclusion and exclusion criteria, our sample comprised 56 333 patients. Treatment delay was defined as initiation of treatment more than 6 weeks after CRC diagnosis, based on data from other studies showing significantly higher mortality in patients treated after that period [22, 23]. Our sample was further divided into patients who did not experience treatment delays (*n* = 45 056) and those who did (*n* = 11 277) (Figure 1).

**FIGURE 1:**
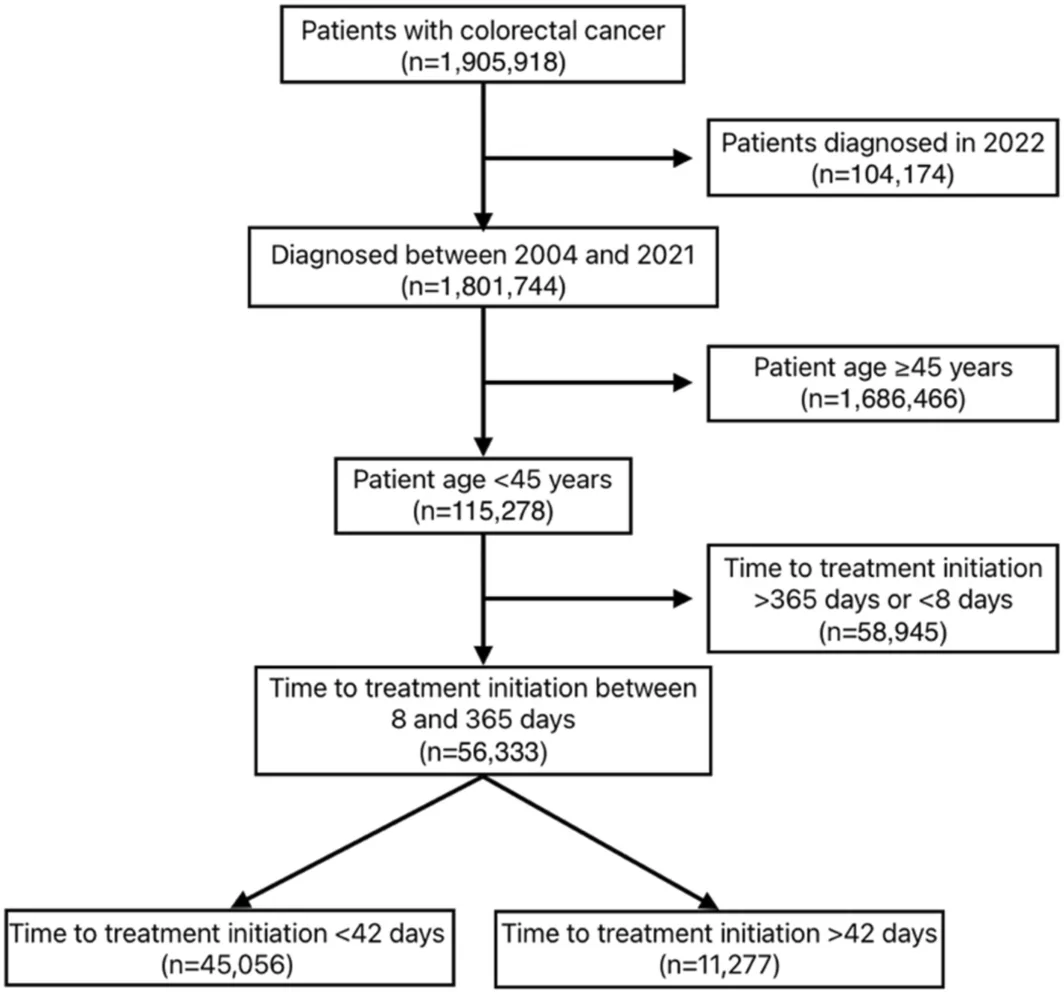
Flow diagram of cohort selection.

### 2.3 Variables of Interest

Our variables of interest included sex, race, ethnicity, setting, literacy, insurance, income, comorbidity burden, and CRC stage. Literacy was gauged using the percentage of high school nongraduates in the patient's residential ZIP code. Income was gauged using the median household income in the patient's residential ZIP code. We utilized the Charlson–Deyo Score as a surrogate for comorbidity burden. Our primary outcome was all‐cause mortality. We assessed this using 5‐year, 10‐year, and overall mortality. The NCDB PUF reports the elapsed months between diagnosis and the date of last contact/death.

### 2.4 Statistical Analysis

For descriptive statistics, we reported the mean age and standard deviation for each cohort, as well as the mean number of days to treatment initiation and its standard deviation. We reported counts and percentages for sex, race, ethnicity, setting, literacy, insurance, income, comorbidity burden, and CRC stage. We tested the significance of these differences using chi‐squared testing, with *p*‐values < 0.05 considered significant.

We evaluated the average annual percentage change (AAPC) in annual mean TTI from 2004 to 2021 for patients with EOCRC by fitting an ordinary least‐squares log‐linear regression to obtain the average increase in TTI over this period. Heteroscedasticity‐robust standard errors were calculated with clustering on diagnosis year to account for within‐year correlation across patients. We reported the AAPC with a 95% confidence interval (CI) and a *p*‐value. This was initially done for the overall cohort, and then separate log‐linear models were run by sex, race, ethnicity, setting, literacy, insurance, income, comorbidity burden, and CRC stage. A *p*‐value of less than 0.05 was considered significant.

We ran multiple regression models to assess predictors of treatment delay and TTI disparities. First, we performed a logistic regression with a treatment delay of greater than 6 weeks as the dependent variable and sex, race, ethnicity, setting, literacy, insurance, income, comorbidity burden, and CRC stage as predictors. For these independent variables, we used males, whites, non‐Hispanics, metropolitan setting, < 5% high school nongraduates, private insurance, ≥ $74 063 household income, Charlson–Deyo Score 0, and Stage 1 as reference, respectively. We conducted a univariate logistic regression using this model, followed by a multivariate logistic regression to control for confounders. We reported our findings as odds ratios (OR) and adjusted odds ratios (aOR), along with their CI and *p*‐values, for univariate and multivariate logistic regression, respectively. Next, we performed a linear regression with the mean number of days to treatment initiation as the dependent variable and the same independent variables as our logistic regression model. We conducted univariate and multivariable linear regression and reported our findings as coefficients in days (d) and adjusted coefficients in days (ad), respectively, along with their CI and *p*‐values. A *p*‐value of less than 0.05 was considered significant. We illustrated our findings as forest plots for both multivariate regressions.

We performed time‐to‐event analyses by fitting a Cox proportional hazards model to obtain the hazard ratios (HR) for 5‐year, 10‐year, and overall all‐cause mortality in patients who experienced a treatment delay compared to those who did not. For each patient, the time scale was defined as the number of months from the date of initial CRC diagnosis to death from any cause. Death from any cause was treated as the event. Patients who were alive at the end of follow‐up were censored at the date of last known contact or at the end of the study period, whichever occurred first. Censored patients were assumed to be alive and event‐free up to their censoring time. We stratified these by CRC stage and conducted univariate and multivariate Cox regression analyses, controlling for sex, race, ethnicity, setting, literacy, insurance, income, and comorbidity burden. We reported our findings as HR and adjusted hazard ratios (aHR), along with their CI and *p*‐values. Our model met the proportional hazard assumption, which was tested using the Schoenfeld Residuals Test. We assessed overall mortality using the Kaplan–Meier method and compared them using the log‐rank test, which was significant. All statistical analyses were performed using StataNow/MP 18.5 for Mac (StataCorp LLC, Texas, USA).

## 3 Results

### 3.1 Frequencies

Our cohort (Table 1) included 56 333 individuals with a mean age of 38.12 years (SD ±5.38) and an average TTI of 32.16 days (SD ±27.02). The cohort had comparable numbers of males (53.5%, *n* = 30 161) and females (46.5%, *n* = 26 172), with similar rates of treatment delay: 79.81% and 80.17%, respectively. White patients (*n* = 43 993) had the lowest percent with delay, with 18.8% experiencing treatment delay, whereas all other races had a higher rate of treatment delay, with American Indian/Alaska Native (AIAN) patients (*n* = 330) having the highest delay proportion at 29.09% (Table 1). Hispanic individuals had a higher rate of treatment delay (27.39%) compared to non‐Hispanic individuals (19.17%).

**TABLE 1:** Frequencies and trends of treatment delays in patients with EOCRC.

	No treatment delay (*n* = 45 056)	Treatment delay (*n* = 11 277)	Total	AAPC in TTI
Sex			*p* = 0.289	
Male	24 073 (79.81%)	6088 (20.19%)	30 161	AAPC = 1.44% (95% CI: 1.31–1.58, *p* < 0.001)
Female	20 983 (80.17%)	5189 (19.83%)	26 172	AAPC = 1.57% (95% CI: 1.42–1.71, *p* < 0.001)
Race			*p* < 0.001	
White	35 724 (81.2%)	8269 (18.8%)	43 993	AAPC = 1.56% (95% CI: 1.45–1.67, *p* < 0.001)
Black	5465 (74.03%)	1917 (25.97%)	7382	AAPC = 1.15% (95% CI: 0.85–1.44, *p* < 0.001)
AIAN	234 (70.91%)	96 (29.09%)	330	AAPC = 1.12% (95% CI: −0.43–2.7, *p* = 0.157)
Asian	2250 (80.21%)	555 (19.97%)	2805	AAPC = 1.43% (95% CI: 0.98–1.88, *p* < 0.001)
Pacific Islander	96 (77.42%)	28 (22.58%)	124	AAPC = 3.10% (95% CI: 0.91–5.33, *p* = 0.006)
Other	788 (75.77%)	252 (24.23%)	1040	AAPC = 1.73% (95% CI: 0.89–2.57, *p* < 0.001)
Ethnicity			*p* < 0.001	
Non‐Hispanic	38 857 (80.83%)	9214 (19.17%)	48 071	AAPC = 1.40% (95% CI: 1.29–1.51, *p* < 0.001)
Hispanic	4485 (72.61%)	1692 (27.39%)	6177	AAPC = 1.72% (95% CI: 1.39–2.06, *p* < 0.001)
Setting			*p* = 0.266	
Metro	36 647 (80.14%)	9083 (19.86%)	45 730	AAPC = 1.49% (95% CI: 1.38–1.60, *p* < 0.001)
Urban	5484 (79.41%)	1422 (20.59%)	6906	AAPC = 1.95% (95% CI: 1.67–2.23, *p* < 0.001)
Rural	767 (78.99%)	204 (21.01%)	971	AAPC = 1.81% (95% CI: 1.04–2.58, *p* < 0.001)
High school nongraduates in patient's area of residence			*p* < 0.001	
< 5.0%	9043 (84.88%)	1611 (15.12%)	10 654	AAPC = 1.20% (95% CI: 0.99–1.41, *p* < 0.001)
5.0%–9.0%	11 398 (81.76%)	2543 (18.24%)	13 941	AAPC = 1.64% (95% CI: 1.45–1.83, *p* < 0.001)
9.1%–15.2%	10 921 (79.14%)	2878 (20.86%)	13 799	AAPC = 1.49% (95% CI: 1.29–1.69, *p* < 0.001)
≥ 15.3%	8433 (74.99%)	2813 (25.01%)	11 246	AAPC = 1.63% (95% CI: 1.39–1.87, *p* < 0.001)
Insurance type			*p* < 0.001	
Private insurance	33 865 (82.76%)	7056 (17.24%)	40 921	AAPC = 1.50% (95% CI: 1.39–1.61, *p* < 0.001)
Not insured	2569 (73.91%)	907 (26.09%)	3476	AAPC = 1.36% (95% CI: 0.91–1.80, *p* < 0.001)
Medicaid	5807 (72.29%)	2226 (27.71%)	8033	AAPC = 1.51% (95% CI: 1.21–1.80, *p* < 0.001)
Medicare	1328 (72.29%)	509 (27.71%)	1837	AAPC = 1.45% (95% CI: 0.88–2.03, *p* < 0.001)
Other government	627 (74.20%)	218 (25.80%)	845	AAPC = 1.79% (95% CI: 0.95–2.65, *p* < 0.001)
Median household income in patient's area of residence			*p* < 0.001	
≥ $74 063	15 928 (82.47%)	3386 (17.53%)	19 314	AAPC = 1.29% (95% CI: 1.13–1.45, *p* < 0.001)
$57 857–$74 062	9469 (80.14%)	2347 (19.86%)	11 816	AAPC = 1.47% (95% CI: 1.26–1.68, *p* < 0.001)
$46 277–$57 856	8103 (79.47%)	2093 (20.53%)	10 196	AAPC = 1.87% (95% CI: 1.64–2.11, *p* < 0.001)
< $46 277	6193 (75.51%)	1987 (24.29%)	8180	AAPC = 1.63% (95% CI: 1.35–1.90, *p* < 0.001)
Charlson–Deyo score			*p* < 0.001	
0	40 738 (80.37%)	9949 (19.63%)	50 687	AAPC = 1.49% (95% CI: 1.38–1.59, *p* < 0.001)
1	3594 (77.61%)	1037 (22.39%)	4631	AAPC = 1.57% (95% CI: 1.21–1.93, *p* < 0.001)
2	458 (73.4%)	166 (26.6%)	624	AAPC = 1.51% (95% CI: 0.44–2.59, *p* = 0.006)
≥ 3	266 (68.03%)	125 (31.97%)	391	AAPC = 1.21% (95% CI: −0.18–2.61, *p* = 0.089)
Stage			*p* < 0.001	
1	5282 (74.45%)	1813 (25.55%)	7095	AAPC = 2.13% (95% CI: 1.83–2.43, *p* < 0.001)
2	8298 (81.79%)	1847 (18.21%)	10 145	AAPC = 1.75% (95% CI: 1.52–1.98, *p* < 0.001)
3	15 568 (80.88%)	3680 (19.12%)	19 248	AAPC = 1.98% (95% CI: 1.82–2.14, *p* < 0.001)
4	13 445 (82.36%)	2879 (17.64%)	16 324	AAPC = 1.13% (95% CI: 0.94–1.32, *p* < 0.001)

Abbreviations: AAPC, average annual percentage change; AIAN, American Indian/Alaska Native; CI, confidence interval; TTI, time to treatment initiation.

Most of our patients were Metropolitan residents (*n* = 45 730), but all settings had similar rates of treatment delay. Educational attainment was linearly associated with treatment delay, with patients from areas with a higher proportion of high‐school nongraduates having higher rates of delayed treatment. Patients with private insurance (*n* = 40 921) had the lowest delay rate, with 17.24% experiencing treatment delay. In contrast, higher delays were observed among those with Medicaid (*n* = 8033) and Medicare (*n* = 1837), both at 27.71%, and among uninsured patients (*n* = 3476), where 26.09% experienced delay. Patients in the lowest income quartile (< $46 277/year; *n* = 8180) had the highest proportion of delays (24.29%) and these proportions were inversely proportional to rising income: $46 277–$57 856 (*n* = 10 196) had 20.53%, $57 857–$74 062 (*n* = 11 816) had 19.86%, and > $74 063/year (*n* = 19 314) had the lowest at 17.53%, underscoring the impact of socioeconomic disadvantage on timely care.

There was a stepwise association between comorbidity burden and delayed care. Most patients had a CDCC score of 0 (*n* = 50 687) and had the lowest delay percentage of 19.63%. The percentage of patients experiencing delays increased with higher scores: CDCC 1 (*n* = 4631) had 22.39%, CDCC 2 (*n* = 624) had 26.6%, and CDCC ≥ 3 (*n* = 391) had the highest at 31.97%. Stage 1 EOCRC patients (*n* = 7095) had 25.55%, compared to Stage 2 (*n* = 10 145) with 18.21%, Stage 3 (*n* = 19 248) with 19.12%, and Stage 4 (*n* = 16 324), with the lowest delay rate at 17.64%.

### 3.2 Trends

The AAPC showed a statistically significant upward overall trend of 1.50% (95% CI: 1.40–1.60, *p* < 0.001) (Table 1). Females had a slightly higher AAPC of 1.57% (95% CI: 1.42–1.71) compared to males at 1.44% (95% CI: 1.31–1.58), with both trends significant (*p* < 0.001). By race, Pacific Islanders had the steepest rise at 3.10% (95% CI: 0.91–5.33, *p* = 0.006), whereas AIAN had the lowest rise at 1.12% (95% CI: −0.43–2.7, *p* = 0.157). Among ethnic groups, non‐Hispanic individuals had an AAPC of 1.40% (95% CI: 1.29–1.51), while Hispanic individuals showed a slightly higher AAPC of 1.72% (95% CI: 1.39–2.06), with both trends statistically significant (*p* < 0.001).

Urban residents had the highest AAPC at 1.95% (95% CI: 1.67–2.23, *p* < 0.001), followed by rural residents at 1.81% (95% CI: 1.04–2.58, *p* < 0.001) and metropolitan residents at 1.49% (95% CI: 1.38–1.60, *p* < 0.001), suggesting worsening trends in disparities with respect to setting. Our results demonstrate worsening TTI disparities in terms of educational attainment, with patients from areas where fewer than 5% of adults lacked a high school diploma having the lowest AAPC at 1.20% (95% CI: 0.99–1.41, *p* < 0.001) compared to patients from areas with lower education, possibly due to a lack of awareness or systemic inequities. By insurance status, uninsured patients had the lowest AAPC at 1.36% (95% CI: 0.91–1.80, *p* < 0.001), suggesting a slower rate of increase in TTI than among insured patients. These findings suggest a narrowing of disparities in terms of insurance status. Median household income in the patient's residence area, however, showed worsening disparity trends, with those in areas earning ≥ $74 063 having an AAPC of 1.29% (95% CI: 1.13–1.45, *p* < 0.001) compared to an AAPC of 1.63% (95% CI: 1.35–1.90, *p* < 0.001) in the lowest‐income areas (< $46 277).

Stratification by CDCC and stage at diagnosis showed varying AAPC. Notably, patients with a score of ≥ 3 had a nonsignificant AAPC of 1.21% (95% CI: −0.18–2.61, *p* = 0.089), possibly due to a small sample size, whereas Stage 1 EOCRC had the highest subgroup AAPC at 2.13% (95% CI: 1.83–2.43, *p* < 0.001) (Table 1).

### 3.3 Predictors of Treatment Delay

Female sex was significantly associated with treatment delay after adjustment compared to males (aOR = 0.94, 95% CI: 0.90–0.99, *p* = 0.020). All racial and ethnic minorities had higher odds of delayed treatment, except Pacific Islanders (aOR = 1.23, 95% CI: 0.75–2.01, *p* = 0.406), compared to white patients. This was most evident among AIAN patients (aOR = 1.41, 95% CI: 1.05–1.88, *p* = 0.021). Hispanics were significantly associated with higher odds of treatment delay compared to non‐Hispanic individuals (aOR = 1.40, 95% CI: 1.30–1.51, *p* < 0.001).

Setting did not significantly influence the odds of treatment delay; however, a higher proportion of adults without a high school diploma in a patient's area was significantly associated with incrementally increased odds of treatment delay with the highest odds in areas where ≥ 15.3% lacked a diploma (aOR = 1.54, 95% CI: 1.41–1.70, *p* < 0.001), signifying an inverse effect of literacy and treatment delays. Compared to patients with private insurance, all insurance types had significantly higher odds of treatment delay: uninsured individuals with of aOR = 1.54 (95% CI: 1.40–1.69, *p* < 0.001), Medicaid with aOR of 1.70 (95% CI: 1.59–1.81, *p* < 0.001), Medicare with aOR of 1.72 (95% CI: 1.52–1.95, *p* < 0.001), and other government insurance with aOR of 1.59 (95% CI: 1.32–1.92, *p* < 0.001). Patients in lower‐income areas had higher odds of treatment delay than those in areas with median household income ≥ $74 063, but this effect was not significant after adjustment for covariates.

Increasing Charlson–Deyo scores were associated with additive, yet insignificant, odds of treatment delay, except for patients with a score ≥ 3 (aOR = 1.55, 95% CI: 1.20–1.99, *p* = 0.001). Compared with Stage 1, patients with advanced disease had significantly lower odds of treatment delay, indicating that those with more advanced disease at diagnosis were less likely to experience delays in treatment initiation (Table 2 and Figure 2).

**TABLE 2:** Predictors of treatment delay using univariate and multivariate logistic and linear regression.

	Univariate logistic regression	Multivariate logistic regression	Univariate linear regression	Multivariate linear regression
Sex				
Male	(Reference)	(Reference)	(Reference)	(Reference)
Female	OR = 0.98 (95% CI: 0.94–1.02, *p* = 0.289)	OR = 0.94 (95% CI: 0.90–0.99, *p* = 0.02)	*d* = −0.48 (95% CI: −0.93–−0.03, *p* = 0.037)	*d* = −0.84 (95% CI: −1.32–−0.36, *p* = 0.001)
Race				
White	(Reference)	(Reference)	(Reference)	(Reference)
Black	OR = 1.52 (95% CI: 1.43–1.61, *p* < 0.001)	OR = 1.38 (95% CI: 1.28–1.48, *p* < 0.001)	*d* = 4.93 (95% CI: 4.27–5.60, *p* < 0.001)	*d* = 3.94 (95% CI: 3.19–4.68, *p* < 0.001)
AIAN	OR = 1.77 (95% CI: 1.40–2.25, *p* < 0.001)	OR = 1.41 (95% CI: 1.05–1.88, *p* = 0.021)	*d* = 5.91 (95% CI: 2.99–8.83, *p* < 0.001)	*d* = 3.30 (95% CI: 0.07–6.53, *p* = 0.045)
Asian	OR = 1.07 (95% CI: 0.97–1.17, *p* = 0.194)	OR = 1.16 (95% CI: 1.03–1.29, *p* = 0.011)	*d* = 0.46 (95% CI: −0.57–1.49, *p* = 0.378)	*d* = 1.23 (95% CI: 0.12–2.33, *p* = 0.029)
Pacific Islander	OR = 1.26 (95% CI: 0.83–1.92, *p* = 0.283)	OR = 1.23 (95% CI: 0.75–2.01, *p* = 0.406)	*d* = 1.30 (95% CI: −3.45–6.06, *p* = 0.591)	*d* = 1.57 (95% CI: −3.58–6.73, *p* = 0.549)
Other	OR = 1.38 (95% CI: 1.20–1.60, *p* < 0.001)	OR = 1.07 (95% CI: 0.90–1.26, *p* = 0.467)	*d* = 3.63 (95% CI: 1.98–5.29, *p* < 0.001)	*d* = 0.73 (95% CI: −1.05–2.51, *p* = 0.422)
Ethnicity				
Non‐Hispanic	(Reference)	(Reference)	(Reference)	(Reference)
Hispanic	OR = 1.59 (95% CI: 1.50–1.69, *p* < 0.001)	OR = 1.40 (95% CI: 1.30–1.51, *p* < 0.001)	*d* = 5.21 (95% CI: 4.49–5.92, *p* < 0.001)	*d* = 3.89 (95% CI: 3.06–4.72, *p* < 0.001)
Setting				
Metro	(Reference)	(Reference)	(Reference)	(Reference)
Urban	OR = 1.05 (95% CI: 0.98–1.11, *p* = 0.158)	OR = 1.05 (95% CI: 0.98–1.14, *p* = 0.18)	*d* = 0.09 (95% CI: −0.60–0.77, *p* = 0.802)	*d* = 0.50 (95% CI: −0.26–1.26, *p* = 0.194)
Rural	OR = 1.07 (95% CI: 0.92–1.25, *p* = 0.376)	OR = 1.03 (95% CI: 0.86–1.23, *p* = 0.753)	*d* = 0.85 (95% CI: −0.87–2.57, *p* = 0.331)	*d* = 1.30 (95% CI: −0.52–3.11, *p* = 0.161)
High school nongraduates in patient's area of residence				
< 5.0%	(Reference)	(Reference)	(Reference)	(Reference)
5.0%–9.0%	OR = 1.25 (95% CI: 1.17–1.34, *p* < 0.001)	OR = 1.21 (95% CI: 1.12–1.31, *p* < 0.001)	*d* = 2.02 (95% CI: 1.34–2.70, *p* < 0.001)	*d* = 1.44 (95% CI: 0.72–2.16, *p* < 0.001)
9.1%–15.2%	OR = 1.48 (95% CI: 1.38–1.58, *p* < 0.001)	OR = 1.37 (95% CI: 1.26–1.49, *p* < 0.001)	*d* = 3.52 (95% CI: 2.84–4.20, *p* < 0.001)	*d* = 2.47 (95% CI: 1.68–3.25, *p* < 0.001)
≥ 15.3%	OR = 1.87 (95% CI: 1.75–2.00, *p* < 0.001)	OR = 1.54 (95% CI: 1.41–1.70, *p* < 0.001)	*d* = 6.10 (95% CI: 5.39–6.81, *p* < 0.001)	*d* = 3.60 (95% CI: 2.68–4.52, *p* < 0.001)
Insurance type				
Private Insurance	(Reference)	(Reference)	(Reference)	(Reference)
Not Insured	OR = 1.69 (95% CI: 1.56–1.84, *p* < 0.001)	OR = 1.54 (95% CI: 1.40–1.69, *p* < 0.001)	*d* = 5.29 (95% CI: 4.36–6.22, *p* < 0.001)	*d* = 4.44 (95% CI: 3.42–5.45, *p* < 0.001)
Medicaid	OR = 1.84 (95% CI: 1.74–1.94, *p* < 0.001)	OR = 1.70 (95% CI: 1.59–1.81, *p* < 0.001)	*d* = 6.36 (95% CI: 5.72–7.01, *p* < 0.001)	*d* = 5.16 (95% CI: 4.45–5.87, *p* < 0.001)
Medicare	OR = 1.84 (95% CI: 1.66–2.04, *p* < 0.001)	OR = 1.72 (95% CI: 1.52–1.95, *p* < 0.001)	*d* = 6.77 (95% CI: 5.51–8.03, *p* < 0.001)	*d* = 5.88 (95% CI: 4.52–7.23, *p* < 0.001)
Other government	OR = 1.67 (95% CI: 1.43–1.95, *p* < 0.001)	OR = 1.59 (95% CI: 1.32–1.92, *p* < 0.001)	*d* = 5.25 (95% CI: 3.42–7.08, *p* < 0.001)	*d* = 4.30 (95% CI: 2.30–6.30, *p* < 0.001)
Median household incomes in patient's area of residence				
≥ $74 063	(Reference)	(Reference)	(Reference)	(Reference)
$57 857–$74 062	OR = 1.17 (95% CI: 1.10–1.24, *p* < 0.001)	OR = 0.99 (95% CI: 0.92–1.06, *p* = 0.703)	*d* = 1.29 (95% CI: 0.67–1.91, *p* < 0.001)	*d* = −0.30 (95% CI: −0.97–0.37, *p* = 0.38)
$46 277–$57 856	OR = 1.22 (95% CI: 1.14–1.29, *p* < 0.001)	OR = 0.93 (95% CI: 0.86–1.00, *p* = 0.065)	*d* = 1.74 (95% CI: 1.09–2.38, *p* < 0.001)	*d* = −0.75 (95% CI: −1.52–0.01, *p* = 0.053)
< $46 277	OR = 1.51 (95% CI: 1.42–1.61, *p* < 0.001)	OR = 0.98 (95% CI: 0.90–1.07, *p* = 0.67)	*d* = 4.08 (95% CI: 3.38–4.77, *p* < 0.001)	*d* = −0.27 (95% CI: −1.17–0.63, *p* = 0.557)
Charlson–Deyo score				
0	(Reference)	(Reference)	(Reference)	(Reference)
1	OR = 1.18 (95% CI: 1.10–1.27, *p* < 0.001)	OR = 1.09 (95% CI: 1.00–1.18, *p* = 0.057)	*d* = 1.37 (95% CI: 0.56–2.18, *p* = 0.001)	*d* = 0.52 (95% CI: −0.36–1.39, *p* = 0.246)
2	OR = 1.48 (95% CI: 1.24–1.78, *p* < 0.001)	OR = 1.23 (95% CI: 1.00–1.52, *p* = 0.056)	*d* = 5.25 (95% CI: 3.12–7.38, *p* < 0.001)	*d* = 3.62 (95% CI: 1.33–5.90, *p* = 0.002)
≥ 3	OR = 1.92 (95% CI: 1.55–2.38, *p* < 0.001)	OR = 1.55 (95% CI: 1.20–1.99, *p* = 0.001)	*d* = 7.88 (95% CI: 5.19–10.56, *p* < 0.001)	*d* = 5.98 (95% CI: 3.10–8.86, *p* < 0.001)
Stage				
1	(Reference)	(Reference)	(Reference)	(Reference)
2	OR = 0.65 (95% CI: 0.60–0.70, *p* < 0.001)	OR = 0.59 (95% CI: 0.55–0.65, *p* < 0.001)	*d* = −5.31 (95% CI: −6.10–−4.53, *p* < 0.001)	*d* = −5.96 (95% CI: −6.80–−5.11, *p* < 0.001)
3	OR = 0.69 (95% CI: 0.65–0.73, *p* < 0.001)	OR = 0.66 (95% CI: 0.61–0.71, *p* < 0.001)	*d* = −4.83 (95% CI: −5.54–−4.13, *p* < 0.001)	*d* = −5.21 (95% CI: −5.97–−4.45, *p* < 0.001)
4	OR = 0.62 (95% CI: 0.58–0.67, *p* < 0.001)	OR = 0.56 (95% CI: 0.52–0.60, *p* < 0.001)	*d* = −5.57 (95% CI: −6.29–−4.85, *p* < 0.001)	*d* = −6.52 (95% CI: −7.31–−5.74, *p* < 0.001)

Abbreviations: AIAN, American Indian/Alaska Native; CI, confidence interval.

**FIGURE 2:**
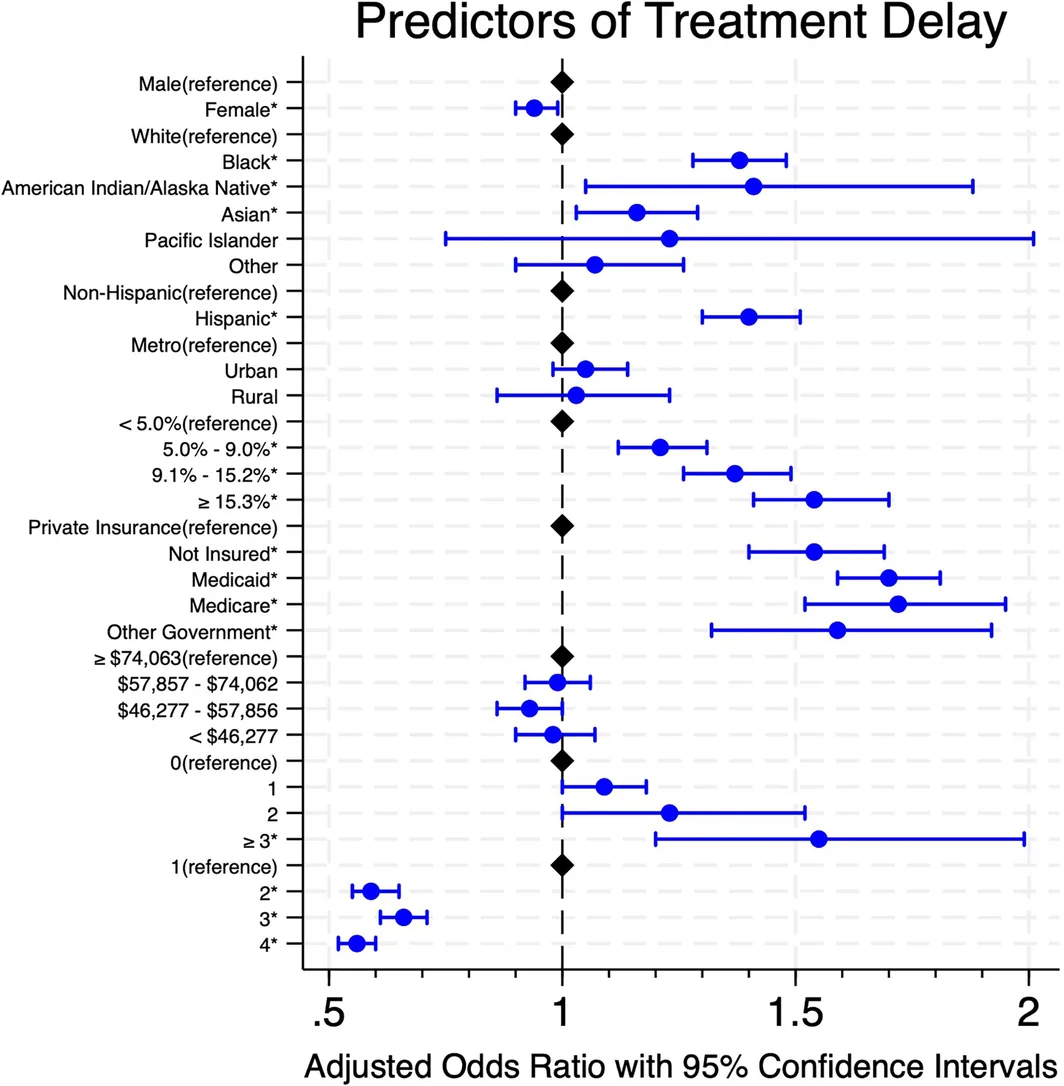
Forest plot of predictors of treatment delay in patients with EOCRC. * demonstrates statistically significant variables.

Linear regression analyses were also conducted, which were consistent with our logistic regression model (Table 2 and Figure 3). Females had a significantly lower mean time to treatment initiation than males (ad = −0.84, 95% CI: −1.32 to −0.36, *p* = 0.001). Similar to our logistic regression model, linear regression showed that all minorities had significantly greater treatment delay than Whites, except for Pacific Islanders (ad = 1.57, 95% CI: −3.58–6.73, *p* = 0.549) with AIAN individuals having the highest mean delay (ad = 3.30, 95% CI: 0.07–6.53, *p* = 0.045). Hispanic individuals also experienced significantly longer days to treatment initiation compared to non‐Hispanics (ad = 3.89, 95% CI: 3.06–4.72, *p* < 0.001).

**FIGURE 3:**
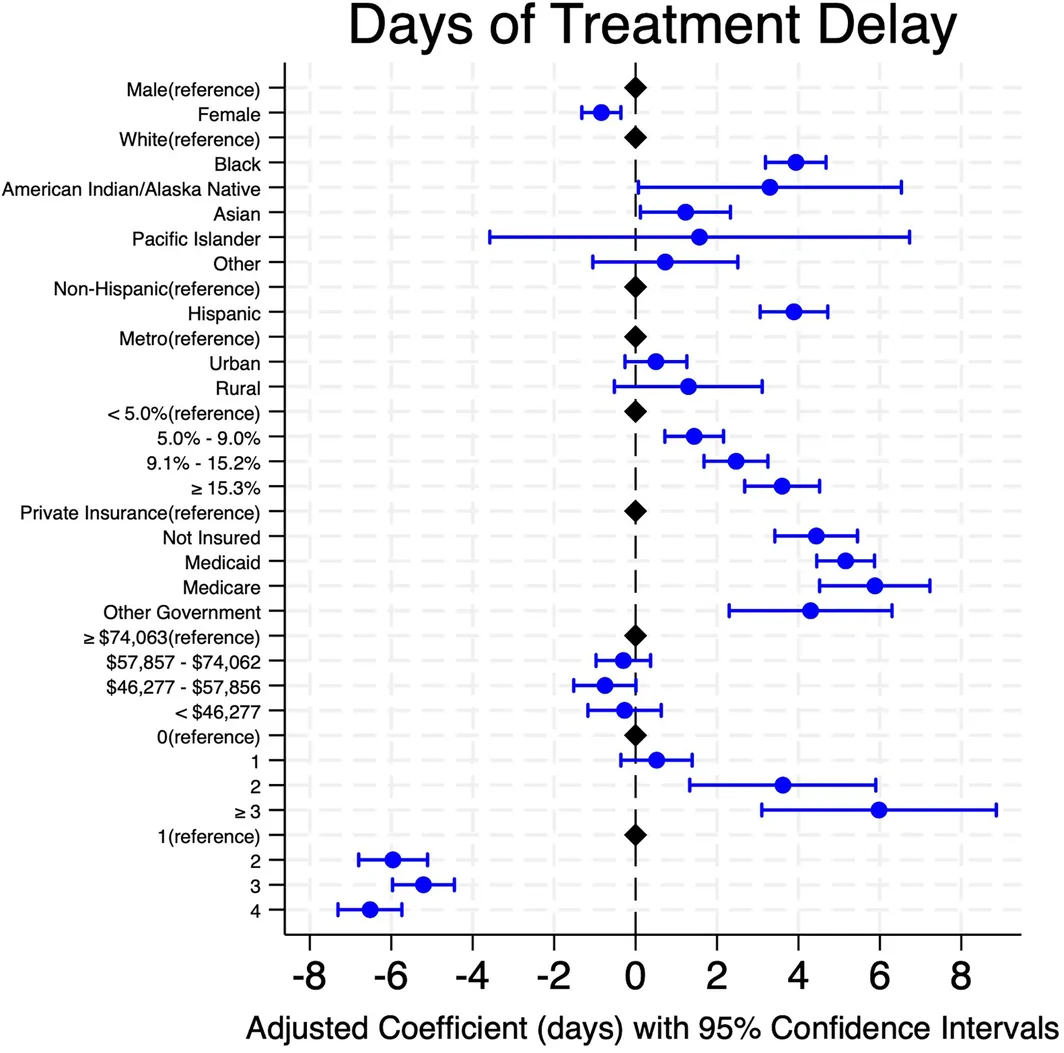
Forest plot of mean delays (days) in patients with EOCRC. * demonstrates statistically significant variables.

The geographic setting did not significantly influence the mean TTI. We noted a strong, dose‐dependent link between lower educational attainment and longer treatment delay; compared to areas with < 5.0% adults lacking a high school diploma, delays were greater in areas with 5.0%–9.0% (ad = 1.44, 95% CI: 0.72–2.16, *p* < 0.001), and 9.1%–15.2% (ad = 2.47, 95% CI: 1.68–3.25, *p* < 0.001), and highest in areas with ≥ 15.3% (ad = 3.60, 95% CI: 2.68–4.52, *p* < 0.001). There was also a significantly longer mean TTI in all insurance groups compared to privately insured patients. Compared to patients from areas with median household income ≥ $74 063, there was no significant difference in mean TTI.

Mean treatment delay increased with higher Charlson–Deyo scores. Compared to score 0, score 1 did not significantly increase after adjustment (ad = 0.52, 95% CI: −0.36–1.39, *p* = 0.246). However, score 2 was associated with significantly longer delays (ad = 3.62, 95% CI: 1.33–5.90, *p* = 0.002), while scores ≥ 3 had the longest significant delays overall (ad = 5.98, 95% CI: 3.10–8.86, *p* < 0.001), implying a lack of urgency in treatment initiation in patients with a higher comorbidity burden. Patients with more advanced cancer stages had significantly shorter treatment delays, reflecting increased clinical urgency (Table 2 and Figure 3).

### 3.4 Mortality

We compared stage‐stratified mortality between patients with and without treatment delay. For 5‐year mortality, treatment delay was linked to higher unadjusted mortality for Stage 1 (HR = 1.26, 95% CI: 1.02–1.56, *p* = 0.032), Stage 2 (HR = 1.68, 95% CI: 1.47–1.93, *p* < 0.001), and Stage 3 (HR = 1.21, 95% CI: 1.12–1.32, *p* < 0.001), but lower unadjusted mortality for Stage 4 (HR = 0.90, 95% CI: 0.85–0.94, *p* < 0.001). After adjustment, only Stage 2 remained significantly associated with increased mortality (aHR = 1.44, 95% CI: 1.23–1.68, *p* < 0.001), and Stage 4 with decreased mortality (aHR = 0.84, 95% CI: 0.79–0.88, *p* < 0.001).

For 10‐year mortality, patients with Stage 1 CRC who experienced treatment delay showed a significant increase in unadjusted mortality (HR = 1.21, 95% CI: 1.02–1.44, *p* = 0.031), with stronger associations in Stage 2 (HR = 1.68, 95% CI: 1.49–1.89, *p* < 0.001) and Stage 3 CRC (HR = 1.23, 95% CI: 1.14–1.32, *p* < 0.001). Treatment delays in patients with Stage 4 CRC were associated with reduced 10‐year mortality (HR = 0.89, 95% CI: 0.85–0.94, *p* < 0.001). After adjusting for covariates, Stage 2 (aHR = 1.49, 95% CI: 1.31–1.70, *p* < 0.001) and Stage 3 (aHR = 1.12, 95% CI: 1.03–1.21, *p* = 0.006) remained independently associated with higher mortality, and Stage 4 with reduced mortality (aHR = 0.84, 95% CI: 0.79–0.89, *p* < 0.001).

Unadjusted overall mortality in patients with treatment delays rose for Stage 1 (HR = 1.27, 95% CI: 1.08–1.49, *p* = 0.004), Stage 2 (HR = 1.65, 95% CI: 1.47–1.85, *p* < 0.001), and Stage 3 (HR = 1.22, 95% CI: 1.13–1.31, *p* < 0.001), whereas Stage 4 was associated with a lower unadjusted overall mortality (HR = 0.89, 95% CI: 0.85–0.94, *p* < 0.001). After adjusting for covariates, Stage 2 (aHR = 1.48, 95% CI: 1.30–1.69, *p* < 0.001) and Stage 3 (aHR = 1.12, 95% CI: 1.03–1.21, *p* = 0.007) remained at significantly increased risk for mortality, while Stage 4 retained a reduced mortality risk (aHR = 0.84, 95% CI: 0.79–0.88, *p* < 0.001) (Table 3 and Figure 4).

**TABLE 3:** Univariate and multivariate Cox regression analysis of 5‐year, 10‐year, and overall mortality compared to patients who did not have treatment delays.

	Univariate HR	95% CI	*p*	Multivariate HR	95% CI	*p*
5‐Year mortality						
Stage 1	1.26	(1.02–1.56)	0.032	1.05	(0.83–1.34)	0.676
Stage 2	1.68	(1.47–1.93)	< 0.001	1.44	(1.23–1.68)	< 0.001
Stage 3	1.21	(1.12–1.32)	< 0.001	1.09	(1.00–1.20)	0.057
Stage 4	0.90	(0.85–0.94)	< 0.001	0.84	(0.79–0.88)	< 0.001
10‐Year mortality						
Stage 1	1.21	(1.02–1.44)	0.031	1.06	(0.87–1.28)	0.58
Stage 2	1.68	(1.49–1.89)	< 0.001	1.49	(1.31–1.70)	< 0.001
Stage 3	1.23	(1.14–1.32)	< 0.001	1.12	(1.03–1.21)	0.006
Stage 4	0.89	(0.85–0.94)	< 0.001	0.84	(0.79–0.89)	< 0.001
Overall mortality						
Stage 1	1.27	(1.08–1.49)	0.004	1.11	(0.92–1.33)	0.273
Stage 2	1.65	(1.47–1.85)	< 0.001	1.48	(1.30–1.69)	< 0.001
Stage 3	1.22	(1.13–1.31)	< 0.001	1.12	(1.03–1.21)	0.007
Stage 4	0.89	(0.85–0.94)	< 0.001	0.84	(0.79–0.88)	< 0.001

Abbreviations: CI, confidence intervals; HR, hazard ratio.

**FIGURE 4:**
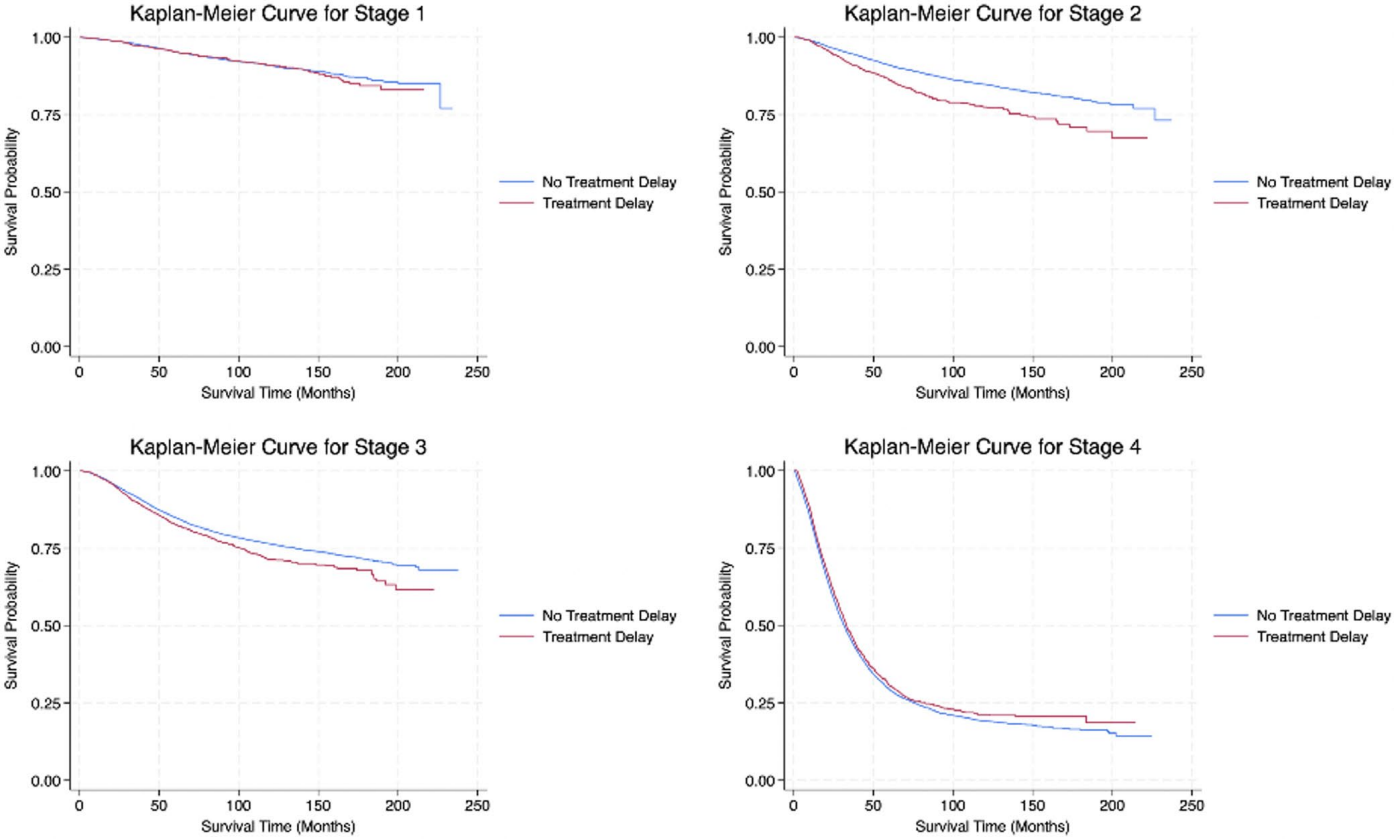
Kaplan–Meier curves for overall survival in patients with EOCRC.

## 4 Discussion

From 1990 to 2019, the number of EOCRC‐related deaths increased from 50 997 to 87 014 per 100 000 population [24]. There have been multiple studies that demonstrate a strong correlation between delays in treatment and the risk of death among patients with CRC. However, data specific to EOCRC have yet to reach definitive conclusions. Our study aimed to uncover trends, predictors, and mortality outcomes associated with treatment delay and TTI across sociodemographic factors and EOCRC stages.

Over the past few decades, TTI for cancer has increased. Bilimoria et al. reported significant increases in TTI for all cancers between 1995 and 2005, with longer delays observed at cancer centers and VA hospitals than at community hospitals [25]. In a study by Patel et al., individuals with EOCRC were found to experience longer symptom duration and delays to diagnosis compared to individuals with late‐onset colorectal cancer [26]. We found that the TTI for patients with EOCRC had an AAPC of 1.50%. This trend was observed across nearly all demographic and clinical subgroups, though the degree of delay varied significantly between different sociodemographic factors. Pacific islanders had a relatively high AAPC, though, given the wide CI, this may be due to the relatively small proportion of these patients in our dataset. Hispanic patients also had a relatively higher rate of change in TTI, suggesting a trend toward worsening disparities in TTI for ethnic minorities.

Rising treatment delays were also evident among patients from nonmetropolitan areas, possibly reflecting limited resources in these settings. Treatment delays were increasingly pronounced among patients from lower‐income and lower‐education communities. This socioeconomic gradient is consistent with prior studies showing that lower socioeconomic status is associated with reduced access to timely cancer care, including longer diagnostic intervals and delayed initiation of treatment [10, 18].

Interestingly, Stage 1 EOCRC had the highest AAPC, which may be due to increased comfort in delaying treatment in these patients with relatively less aggressive tumors. Similar findings were reported by Min et al., who examined five common cancers in females, suggesting that resources should be reallocated to prioritize patients with more advanced disease [27]. Conversely, black patients had a relatively small AAPC, possibly due to increased awareness among clinicians. In a study by Adamson et al., they noted a similar trend in the reduction of disparities for advanced care, possibly due to Medicaid expansion [28].

In our study, we noted multiple independent predictors of delayed TTI in patients with EOCRC. Racial and ethnic disparities were especially prominent; Blacks, Asians, and AIAN had higher odds of experiencing treatment delays compared to White patients. Hispanic patients also had higher odds compared to non‐Hispanic patients. These findings reflect current literature, which highlights the disproportionate systemic barriers racial and ethnic minorities face to timely cancer care. These include differences in referral patterns and provider availability [29]. Such disparities may be further compounded by implicit bias in clinical decision‐making, language barriers, and geographic maldistribution of oncology services, all of which disproportionately affect minoritized communities.

Insurance status emerged as one of the strongest predictors of delay. Compared to privately insured patients, those uninsured, on Medicaid, or Medicare had 54%, 70%, and 72% higher odds of treatment delay, respectively. These findings mirror data from Yabroff et al., who reported that public or no insurance was linked to longer diagnostic and treatment intervals in patients with cancer [30]. Barriers such as network restrictions, authorization delays, and lower healthcare navigation support are well‐documented contributors. Community‐level education, used as a proxy for socioeconomic status, showed a dose‐dependent relationship with treatment delay, both in terms of odds of experiencing delayed treatment and the degree to which treatment was delayed. This may be due to a direct correlation between lower education and limited health literacy, which can lead to weaker provider advocacy and underutilization of healthcare services, as seen in prior studies [31, 32]. Interestingly, area‐level income and urban/rural residence were not significant predictors in adjusted models, suggesting that educational attainment and insurance coverage more directly influence access.

Clinical characteristics also contributed to predicting treatment delay. Patients with higher Charlson–Deyo comorbidity scores had increased odds and amount of delay. This is an expected finding, as comorbidities often delay treatment initiation due to coordination demands and perioperative risk assessments [33]. Conversely, patients with more advanced‐stage disease had significantly lower odds of delay. Specifically, Stage 4 patients had 44% lower odds of delay than Stage 1 patients. This is reflected in the current literature, which demonstrates a shorter interval between diagnosis and treatment in late‐stage cancers [34]. These findings may reflect increased urgency in managing advanced disease or the use of differential clinical pathways for patients with later‐stage diagnoses.

Our analysis of TTI in EOCRC revealed stage‐specific variation in how delays impact mortality. Treatment delays were significantly associated with increased mortality in Stages 2 and 3 disease, but not in Stage 1 or Stage 4 disease. In Stage 2, delay was associated with a 44% higher risk of 5‐year mortality and a 49% higher risk of 10‐year mortality. Similarly, for Stage 3, 10‐year mortality was modestly increased. Cone et al. reported similar results regarding Stage 3 CRC [13]. These findings support the notion that delayed treatment initiation in locally advanced disease can worsen prognosis, particularly when curative intent remains viable. This is noteworthy, as the rate of patients presenting with late‐stage EOCRC has been increasing. A study using data from the SEER program found that from 2000 to 2002 to 2014–2016, the incidence of late‐stage colon adenocarcinoma increased by 49% in people aged 30–39 and by 133% in those aged 20–29 with rectal adenocarcinoma [35].

In contrast, no significant association between delay and mortality was observed in Stage 1 disease. We suspect this is because early‐stage tumors may be biologically indolent, and short delays are unlikely to affect outcomes meaningfully. This is consistent with data from a study by Hanna et al., which suggested that brief delays in early‐stage cancers do not carry the same mortality risks as in later stages [36]. Notably, treatment delays in Stage 4 disease were paradoxically associated with lower mortality. These findings may reflect residual confounding or survivor bias and should be interpreted cautiously. It is not an unexpected finding, as patients with rapidly progressive, symptomatic disease are triaged for faster treatment but may have poorer outcomes regardless of timing. Because of this, many studies evaluating treatment delay and outcomes specifically exclude Stage 4 disease. However, it is important to recognize that patients with EOCRC are more likely to present with advanced‐stage disease at diagnosis, often due to delayed recognition of symptoms and aggressive tumor biology [37, 38].

Advanced‐stage disease is also more aggressive and challenging to treat effectively, potentially explaining why shorter TTI in Stage 4 EOCRC did not improve overall mortality. These results underscore the complexity of TTI and outcomes in EOCRC and highlight the need for stage‐specific and disease‐specific considerations in treatment strategies. It also calls for greater awareness and recognition of EOCRC symptoms by patients and their clinicians. A higher index of suspicion for EOCRC among our population can help us reduce the rate of advanced‐stage disease at initial presentation, enabling us to detect the malignancy at an earlier, more treatable stage. This may also have implications for reassessing our screening approach for this patient population.

We identified several limitations with our study, mostly pertaining to the dataset we used. Firstly, we could not identify the reason for the delay, which could introduce bias. There may have been confounders and competing risks contributing to delays that were not recorded. The COVID‐19 pandemic may have skewed our findings due to delays caused by hospital Standard Operating Procedures. Furthermore, there is evidence to suggest widening of disparities during the pandemic, particularly regarding cancer care [39]. This may have compounded the treatment disparities noted in our study, though this may be beneficial for clinicians and policymakers, as it paints a scenario worse than expected and allows them to make precocious interventions. Notably, the NCDB PUF obtains its records from Commission on Cancer‐accredited facilities, potentially excluding smaller centers that serve minorities. This may explain the underrepresentation of certain minorities and the lack of power, resulting in wider CI for certain predictors of treatment delay that might otherwise have been significant [40]. Though the NCDB follows standardized, high‐quality data‐collection techniques, coding errors remain a concern, as they do in all datasets. We attempted to address this by excluding data points that might raise validity concerns. The NCDB records hospital‐based rather than population‐based data, restricting its generalizability. Additionally, PUF reports all‐cause mortality rather than cancer‐specific mortality. This limitation is even more noteworthy in EOCRC, as younger patients with a history of malignancies are more likely to have a noncancer‐related cause of death [41]. Treatment delay was modeled as a fixed exposure defined after diagnosis rather than as a time‐dependent variable, so that patients could be classified as delayed only after they had survived long enough to meet the 6‐week definition. This design is susceptible to immortal time bias across all stage‐specific models, not only in Stage 4, and tends to make the delayed group appear healthier than it truly is. In Stage 4, where early mortality is more common, this mechanism may explain, in whole or in part, the observed survival advantage associated with delay; this finding should therefore be regarded as hypothesis‐generating rather than as evidence that delaying treatment improves survival. In Stages 2 and 3, immortal time bias may also have attenuated the observed adverse association between treatment delay and mortality. Accordingly, the reported HR should be interpreted as associative rather than causal and may underestimate the true magnitude of the association.

Our study demonstrates that treatment delays are increasingly common over time and disproportionately affect individuals with socioeconomic disadvantage, including those with nonprivate insurance, lower educational attainment, and minority racial/ethnic backgrounds, which may reflect growing strain on the healthcare system due to heightened cancer screening, resulting in a higher prevalence with limited oncological service capacity. Importantly, delayed time to treatment initiation was associated with increased mortality in Stages 2 and 3 disease, highlighting the clinical significance of timely care in potentially curable patients. While timely treatment is critical, the impact of treatment delay was less pronounced in early‐stage and metastatic disease. This highlights the importance of aligning treatment strategies with overall mortality outcomes and patient values. In Stage 4 EOCRC, where disease severity often drives outcomes more than timing, supportive care and goals‐of‐care discussions should be prioritized when appropriate. To our knowledge, this is one of the few studies to assess the impact of TTI delay in EOCRC across all disease stages. Our findings underscore the need for tailored interventions to address treatment delays among high‐risk populations, particularly those with socioeconomic barriers, to improve outcomes in EOCRC. Efforts should focus on optimizing care delivery after diagnosis and prioritizing timely treatment initiation in patients most likely to benefit.

## Funding

The authors have nothing to report.

## Ethics Statement

Institutional review board exemption was granted for this study, as it does not directly involve humans.

## Conflicts of Interest

The authors declare no conflicts of interest.

## Data Availability

All data were derived from the National Cancer Database, which is publicly available through an application process for investigators associated with the Commission on Cancer‐accredited cancer programs of the American College of Surgeons (https://www.facs.org/quality‐programs/cancer/ncdb).

## References

[1] G. Roshandel , F. Ghasemi‐Kebria , and R. Malekzadeh , “Colorectal Cancer: Epidemiology, Risk Factors, and Prevention,” Cancers 16, no. 8 (2024): 1530. 10.3390/cancers16081530 PMC11049480 38672612

[2] F. A. Sinicrope , “Increasing Incidence of Early‐Onset Colorectal Cancer,” New England Journal of Medicine 386, no. 16 (2022): 1547–1558, 10.1056/NEJMra2200869. 35443109

[3] R. L. Siegel , A. N. Giaquinto , and A. Jemal , “Cancer Statistics, 2024,” CA: A Cancer Journal for Clinicians 74, no. 1 (2024): 12–49, 10.3322/caac.21820. 38230766

[4] R. L. Siegel , S. A. Fedewa , W. F. Anderson , et al., “Colorectal Cancer Incidence Patterns in the United States, 1974–2013,” Journal of the National Cancer Institute 109, no. 8 (2017): djw322, 10.1093/jnci/djw322. PMC6059239 28376186

[5] A. Bhandari , M. Woodhouse , and S. Gupta , “Colorectal Cancer Is a Leading Cause of Cancer Incidence and Mortality Among Adults Younger Than 50 Years in the USA: A SEER‐Based Analysis With Comparison to Other Young‐Onset Cancers,” Journal of Investigative Medicine 65, no. 2 (2017): 311–315, 10.1136/jim-2016-000229. PMC5564445 27864324

[6] R. Laskar , C. Qu , J. Huyghe , et al., “Genome‐Wide Association Studies and Mendelian Randomization Analyses Provide Insights Into the Causes of Early‐Onset Colorectal Cancer,” Annals of Oncology 35, no. 6 (2024): 523–536, 10.1016/j.annonc.2024.02.008. PMC11213623 38408508

[7] B. Nelson and W. Faquin , “Decoding the Mysterious Surge in Early‐Onset Colorectal Cancers,” Cancer Cytopathology 133, no. 8 (2025): e70025, 10.1002/cncy.70032. 40762631

[8] S. Li , J. I. Keenan , I. C. Shaw , and F. A. Frizelle , “Could Microplastics Be a Driver for Early Onset Colorectal Cancer?,” Cancers 15, no. 13 (2023): 3323, 10.3390/cancers15133323. PMC10340669 37444433

[9] A. Farooq , A. R. Keehn , Y. Xu , et al., “Patient and Disease Characteristics, Treatment Practices and Oncologic Outcomes Among Patients With Colorectal Cancer: A Population‐Based Analysis,” Canadian Journal of Surgery 66, no. 1 (2023): E71–E78, 10.1503/cjs.024320. PMC9943546 36792127

[10] S. P. Ang , E. Lee , J. E. Chia , et al., “Time‐to‐Treatment Initiation and Its Effect on All‐Cause Mortality: Insights From the Surveillance, Epidemiology, and End Results Database,” World Journal of Oncology 16, no. 3 (2025): 286–294, 10.14740/wjon2584. PMC12185122 40556964

[11] M. A. Sekeres , P. Elson , M. E. Kalaycio , et al., “Time From Diagnosis to Treatment Initiation Predicts Survival in Younger, but Not Older, Acute Myeloid Leukemia Patients,” Blood 113, no. 1 (2009): 28–36, 10.1182/blood-2008-05-157065. PMC2614639 18827183

[12] G. J. Dafflisio , M. Wang , X. Wang , et al., “Time to Treatment Initiation for the 30 Most Prevalent Cancer Types: Trends and Predictors of Change,” JCO Oncology Practice 22 (2025): 141–150, 10.1200/op.23.00614. 40393016

[13] E. B. Cone , M. Marchese , M. Paciotti , et al., “Assessment of Time‐To‐Treatment Initiation and Survival in a Cohort of Patients With Common Cancers,” JAMA Network Open 3, no. 12 (2020): e2030072, 10.1001/jamanetworkopen.2020.30072. PMC7737088 33315115

[14] T. M. Ko , K. N. Laraia , H. R. Alexander , et al., “Low Neighborhood Socioeconomic Status Is Associated With Poor Outcomes in Young Adults With Colorectal Cancer,” Surgery 176, no. 3 (2024): 626–632, 10.1016/j.surg.2024.05.031. 38972769

[15] C. C. Beauharnais , A. Crawford , J. E. Springer , et al., “Racial Disparities in Survival of Early Onset Colon Cancer (Age < 50): A Matched NCDB Analysis,” American Journal of Surgery 228 (2024): 141–145, 10.1016/j.amjsurg.2023.08.025. 37718168

[16] N. S. Wagle , L. Nogueira , T. P. Devasia , et al., “Cancer Treatment and Survivorship Statistics, 2025,” CA: A Cancer Journal for Clinicians 75, no. 4 (2025): 308–340, 10.3322/caac.70011. PMC12223361 40445120

[17] R. A. Smith , K. S. Andrews , D. Brooks , et al., “Cancer Screening in the United States, 2017: A Review of Current American Cancer Society Guidelines and Current Issues in Cancer Screening,” CA: A Cancer Journal for Clinicians 67, no. 2 (2017): 100–121, 10.3322/caac.21392. 28170086

[18] K. Mallin , A. Browner , B. Palis , et al., “Incident Cases Captured in the National Cancer Database Compared With Those in U.S. Population Based Central Cancer Registries in 2012–2014,” Annals of Surgical Oncology 26, no. 6 (2019): 1604–1612, 10.1245/s10434-019-07213-1. 30737668

[19] K. Y. Bilimoria , A. K. Stewart , D. P. Winchester , and C. Y. Ko , “The National Cancer Data Base: A Powerful Initiative to Improve Cancer Care in the United States,” Annals of Surgical Oncology 15, no. 3 (2008): 683–690, 10.1245/s10434-007-9747-3. PMC2234447 18183467

[20] C. T. Murphy , T. J. Galloway , E. A. Handorf , et al., “Increasing Time to Treatment Initiation for Head and Neck Cancer: An Analysis of the National Cancer Database,” Cancer 121, no. 8 (2015): 1204–1213, 10.1002/cncr.29191. 25490875

[21] T. R. Cushman , B. Jones , D. Akhavan , et al., “The Effects of Time to Treatment Initiation for Patients With Non‐Small‐Cell Lung Cancer in the United States,” Clinical Lung Cancer 22, no. 1 (2021): e84–e97, 10.1016/j.cllc.2020.09.004. 33039348

[22] C. M. Stuart , N. M. Mott , M. R. Bronsert , et al., “The Association Between Sociodemographic Factors and Delays to Minimally Invasive Surgery for Stage IA‐IIIA Non‐Small Cell Lung Cancer,” Annals of Thoracic Surgery 119, no. 5 (2025): 1082–1091, 10.1016/j.athoracsur.2024.12.009. 39725251

[23] N. F. Kanarek , C. M. Hooker , L. Mathieu , et al., “Survival After Community Diagnosis of Early‐Stage Non‐Small Cell Lung Cancer,” American Journal of Medicine 127, no. 5 (2014): 443–449, 10.1016/j.amjmed.2013.12.023. PMC4601577 24486286

[24] H. Pan , Z. Zhao , Y. Deng , et al., “The Global, Regional, and National Early‐Onset Colorectal Cancer Burden and Trends From 1990 to 2019: Results From the Global Burden of Disease Study 2019,” BMC Public Health 22, no. 1 (2022): 1896, 10.1186/s12889-022-14274-7. PMC9555189 36221047

[25] K. Y. Bilimoria , C. Y. Ko , J. S. Tomlinson , et al., “Wait Times for Cancer Surgery in the United States,” Annals of Surgery 253, no. 4 (2011): 779–785, 10.1097/sla.0b013e318211cc0f. 21475020

[26] S. G. Patel , C. C. Murphy , C. H. Lieu , and H. Hampel , “Chapter One—Early Age Onset Colorectal Cancer,” in Advances in Cancer Research, vol. 151, ed. F. G. Berger and C. R. Boland (Academic Press, 2021), 1–37. 10.1016/bs.acr.2021.03.001 34148611

[27] Y. Min , Z. Liu , R. Huang , et al., “Survival Outcomes Following Treatment Delays Among Patients With Early‐Stage Female Cancers: A Nationwide Study,” Journal of Translational Medicine 20, no. 1 (2022): 560, 10.1186/s12967-022-03719-7. PMC9719121 36463201

[28] B. J. S. Adamson , A. B. Cohen , C. P. Gross , et al., “ACA Medicaid Expansion Association With Racial Disparity Reductions in Timely Cancer Treatment,” American Journal of Managed Care 27, no. 7 (2021): 274–281, 10.37765/ajmc.2021.88700. 34314116

[29] H. M. Sineshaw , K. Ng , W. D. Flanders , O. W. Brawley , and A. Jemal , “Factors That Contribute to Differences in Survival of Black vs White Patients With Colorectal Cancer,” Gastroenterology 154, no. 4 (2018): 906–915.e7, 10.1053/j.gastro.2017.11.005. PMC5847437 29146523

[30] K. R. Yabroff , K. Reeder‐Hayes , J. Zhao , et al., “Health Insurance Coverage Disruptions and Cancer Care and Outcomes: Systematic Review of Published Research,” Journal of the National Cancer Institute 112, no. 7 (2020): 671–687, 10.1093/jnci/djaa048. PMC7357319 32337585

[31] B. F. Yagi , J. E. Luster , A. M. Scherer , M. R. Farron , J. E. Smith , and R. Tipirneni , “Association of Health Insurance Literacy With Health Care Utilization: A Systematic Review,” Journal of General Internal Medicine 37, no. 2 (2022): 375–389, 10.1007/s11606-021-06819-0. PMC8141365 34027605

[32] T. C. Davis , M. V. Williams , E. Marin , R. M. Parker , and J. Glass , “Health Literacy and Cancer Communication,” CA: A Cancer Journal for Clinicians 52, no. 3 (2002): 134–149, 10.3322/canjclin.52.3.134. 12018928

[33] D. Sarfati , B. Koczwara , and C. Jackson , “The Impact of Comorbidity on Cancer and Its Treatment,” CA: A Cancer Journal for Clinicians 66, no. 4 (2016): 337–350, 10.3322/caac.21342. 26891458

[34] F. Macià , J. Pumarega , M. Gallén , and M. Porta , “Time From (Clinical or Certainty) Diagnosis to Treatment Onset in Cancer Patients: The Choice of Diagnostic Date Strongly Influences Differences in Therapeutic Delay by Tumor Site and Stage,” Journal of Clinical Epidemiology 66, no. 8 (2013): 928–939, 10.1016/j.jclinepi.2012.12.018. 23810030

[35] E. M. Montminy , M. Zhou , L. Maniscalco , et al., “Shifts in the Proportion of Distant Stage Early‐Onset Colorectal Adenocarcinoma in the United States,” Cancer Epidemiology, Biomarkers & Prevention 31, no. 2 (2022): 334–341, 10.1158/1055-9965.epi-21-0611. 35082122

[36] T. P. Hanna , W. D. King , S. Thibodeau , et al., “Mortality due to Cancer Treatment Delay: Systematic Review and Meta‐Analysis,” BMJ 371 (2020): m4087, 10.1136/bmj.m4087. PMC7610021 33148535

[37] C.‐K. Liao , Y. J. Hsu , Y. J. Chern , et al., “Differences in Characteristics and Outcomes Between Early‐Onset Colorectal Cancer and Late‐Onset Colorectal Cancers,” European Journal of Surgical Oncology 50, no. 12 (2024): 108687, 10.1016/j.ejso.2024.108687. 39288563

[38] S. G. Patel , F. P. May , J. C. Anderson , et al., “Updates on Age to Start and Stop Colorectal Cancer Screening: Recommendations From the U.S. Multi‐Society Task Force on Colorectal Cancer,” American Journal of Gastroenterology 117, no. 1 (2022): 57–69, 10.14309/ajg.0000000000001548. 34962727

[39] J. Y. Islam , C. A. Hathaway , E. Hume , et al., “Racial and Ethnic Inequities in Cancer Care Continuity During the COVID‐19 Pandemic Among Those With SARS‐CoV‐2,” JAMA Network Open 7, no. 5 (2024): e2412050, 10.1001/jamanetworkopen.2024.12050. PMC11107297 38767916

[40] Y. Satpathy , P. Nam , M. Moldovan , et al., “Comparison of Capture Rates of the National Cancer Database Across Race and Ethnicity,” JAMA Network Open 6, no. 12 (2023): e2350237, 10.1001/jamanetworkopen.2023.50237. PMC10753391 38150248

[41] N. G. Zaorsky , T. M. Churilla , B. L. Egleston , et al., “Causes of Death Among Cancer Patients,” Annals of Oncology 28, no. 2 (2017): 400–407, 10.1093/annonc/mdw604. PMC5834100 27831506

